# Systemic Medications as Triggers of Acute Angle-Closure Glaucoma: A Narrative Review

**DOI:** 10.3390/jcm15155834

**Published:** 2026-07-26

**Authors:** Motazz Alarfaj, Jumanah Qedair

**Affiliations:** Department of Ophthalmology, College of Medicine, Imam Abdulrahman Bin Faisal University, Dammam 34212, Saudi Arabia

**Keywords:** acute angle-closure glaucoma, drug-induced glaucoma, systemic medications, sulfonamides, topiramate, antidepressants, anticholinergics, prescribing safety

## Abstract

**Background:** Acute angle-closure glaucoma (AACG) is an emergency requiring rapid intervention to prevent irreversible optic nerve damage and permanent vision loss. **Methods**: This narrative review synthesizes the systemic medication classes most implicated in medication-induced AACG, outlining their underlying pathophysiological mechanisms, typical timing of onset, and practical risk-reduction strategies. Evidence was collected through targeted searches of the PubMed, Google Scholar, Cochrane Library, and Web of Science databases, focusing on major review articles, pharmacoepidemiologic studies, and clinical consensus guidelines. Source selection and data synthesis followed a narrative approach without formal risk of bias assessment or meta-analysis. **Results**: The literature consistently implicates sulfonamide derivatives (mainly topiramate), serotonergic antidepressants and triptans, potent systemic anticholinergics, and adrenergic decongestants. Sulfonamides typically precipitate bilateral, non-pupillary-block AACG via ciliochoroidal effusion, secondary anterior displacement of the lens-iris diaphragm, and an acute myopic shift. Conversely, serotonergic, anticholinergic, and adrenergic agents typically trigger classic pupillary-block AACG in anatomically predisposed eyes with pre-existing narrow angles. Adverse events predominantly occur shortly after treatment initiation or dose escalation. Management relies on prompt medication discontinuation and rapid intraocular pressure reduction. Importantly, cycloplegics are preferred over miotics in effusion-induced cases. Given the rarity of these events, brief patient education represents a pragmatic and scalable approach, although its direct clinical effectiveness in reducing event rates has not yet been formally established. **Conclusions**: A select group of widely prescribed systemic medications are associated with most cases of medication-induced AACG. Improving clinician awareness, providing brief patient counseling on warning symptoms, and ensuring prompt ophthalmic evaluation represent the most practical currently available risk-reduction strategies to prevent vision loss.

## 1. Introduction

As a severe, acute subtype of glaucoma, acute angle-closure glaucoma (AACG) causes sudden elevation of intraocular pressure (IOP) and represents an ophthalmic emergency requiring immediate intervention to prevent irreversible optic nerve damage [1,2,3]. The resulting clinical presentation is acute with sudden ocular pain, severe headache, decreased visual acuity, and/or systemic autonomic symptoms, such as nausea and vomiting secondary to the acute pressure spike [4].

Primary angle-closure disease is especially prevalent in Asian populations and is associated with older age, female sex, hyperopia, shallow anterior chambers, and certain ethnic backgrounds [2,3,5]. In addition to primary mechanisms, acute angle closure may be triggered by medications that induce pupillary dilation or uveal effusion in susceptible eyes [6,7,8,9,10,11,12,13,14].

Pharmacoepidemiologic studies and the case-based literature suggest that a relatively small group of systemic medication classes account for a substantial proportion of reported medication-induced AACG events [6,7,8,9,15,16,17,18]. Because many of these agents are prescribed by neurologists, psychiatrists, internists, urologists, and other non-ophthalmic clinicians, prevention depends on practical cross-specialty measures rather than complex ophthalmic algorithms [19,20].

While the topic of medication-induced AACG has been investigated in previously published reviews, this current review bridges the remaining literature gaps by synthesizing the most up-to-date, population-level risk data available in current medical literature and shifting the prevention strategy away from universal baseline screening toward a practical counseling model designed for cross-specialty prescribers. Accordingly, this narrative review synthesizes current evidence on the systemic medication classes most commonly implicated in AACG, outlines their mechanisms and clinical patterns, and proposes a practical prescribing message centered on patient education and urgent ophthalmic review if symptoms occur.

## 2. Materials and Methods

### 2.1. Search Strategy

A targeted narrative literature search was performed across the PubMed, Google Scholar, Cochrane library, and Web of Science databases from database inception through May 2026. The search strategy was developed using a combination of Medical Subject Headings (MeSH) terms and free-text keywords, including (“acute angle-closure glaucoma” OR “AACG”) AND (“drug-induced” OR “iatrogenic” OR “topiramate” OR “sulfonamide” OR “antidepressant” OR “triptan” OR “anticholinergic” OR “alpha-blocker” OR “benign prostatic hyperplasia” OR “decongestant”).

### 2.2. Study Selection and Data Synthesis

Literature was screened for clinical relevance to medication-associated angle closure. Ultimately, forty-three core publications comprising landmark reviews, pharmacoepidemiologic studies, key case series and reports, and major international glaucoma guidelines were selected for synthesis based on their clinical value and direct relevance to the review objectives. Studies were excluded if they involved IOP elevation caused by trauma, intraocular tumors, neovascularization, or uveitis unrelated to medication-induced uveal effusion. Records were also excluded if they lacked diagnostic confirmation of AACG or failed to isolate the specific suspected pharmacological agent. Formal risk of bias assessment, meta-analysis, and uniform tabulation of medication-specific incidence estimates were not performed given the narrative scope of this review, the heterogeneity of available evidence, and the reliance on observational and case-based literature.

## 3. Results

### 3.1. Pathophysiology of Medication-Induced Acute Angle-Closure Glaucoma (AACG)

The pathophysiology of medication-induced AACG is hypothesized to be mediated through two main mechanisms, each carrying unique therapeutic implications. The first mechanism is pupillary-block AACG, which occurs when resistance to aqueous flow from the posterior to the anterior chamber increases, producing iris bombe and peripheral angle closure. Medications that induce intermediate pupil dilation (approximately 6–7 mm), particularly systemic anticholinergic and adrenergic agents, and some serotonergic medications, carry the greatest risk in anatomically predisposed eyes; both minimal and fully pharmacologic mydriasis appear to confer less risk than a partially dilated intermediate pupil [2,3,6,7,8,9,16,17,18].

On the other hand, non-pupillary-block AACG is classically triggered with sulfonamide-derived medications, such as topiramate, in which ciliochoroidal effusion and ciliary body edema push the lens-iris diaphragm anteriorly, leading to bilateral shallow anterior chambers, myopic shift, and circumferential angle closure without iris bombe [6,7,8,9,10,11,12,13,14,21,22]. Distinguishing between these two pathways is clinically vital, as miotics and laser peripheral iridotomy (LPI) are effective for classic pupillary-block AACG but can worsen effusion-related non-pupillary-block AACG, where cycloplegia, corticosteroids, and prompt medication withdrawal are the core management strategies [23,24].

### 3.2. Systemic Medications Implicated in AACG

Many systemic medication classes have been associated with AACG, but the most consistently implicated groups include sulfonamide-derived agents, serotonergic antidepressants and tricyclics, triptans, anticholinergics, adrenergic decongestants and sympathomimetics, and some alpha blockers. The strength of the clinical evidence associating these systemic medication classes to iatrogenic AACG varies significantly across the literature. While certain classes like serotonergic antidepressants and alpha-blockers are substantiated by large pharmacoepidemiologic databases, other associations, such as those involving triptans, remain supported primarily by case reports and safety databases. This variation dictates the clinical confidence with which pre-treatment risks can be evaluated (Table 1).

#### 3.2.1. Sulfonamide-Derived Agents

Sulfonamide-derived agents, led by topiramate and zonisamide, are the most comprehensively documented medication class in the AACG literature [10,11,12,13,14,22]. Their mechanism differs fundamentally from that of other implicated classes. Rather than inducing mydriasis, they cause ciliochoroidal effusion and ciliary body edema, which push the lens-iris diaphragm anteriorly and produce bilateral circumferential angle closure with an acute myopic shift of several diopters. Because iris bombe and pupillary block are absent, pilocarpine and LPI are not only ineffective but may be harmful; medication discontinuation, cycloplegia, and topical corticosteroids form the therapeutic cornerstone [25,26]. Episodes typically occur within the first two weeks of treatment or after dose escalation. Importantly, this mechanism does not require pre-existing narrow angles and may occur in anatomically normal eyes, limiting the predictive value of pretreatment gonioscopy for this medication class [10,11,12,13,14,22].

#### 3.2.2. Serotonergic Antidepressants and Tricyclic Antidepressants

Serotonergic antidepressants (SSRIs and SNRIs) and tricyclic antidepressants are widely prescribed and represent a clinically important cause of medication-induced AACG [27]. Population-level studies have documented an increased risk in the period shortly after treatment initiation, predominantly in older patients with anatomically narrow angles [17,18]. The proposed mechanism is thought to involve mild pharmacologic mydriasis, which may be theoretically compounded by serotonergic vascular effects that alter aqueous dynamics, potentially leading to pupillary-block AACG in predisposed eyes. Tricyclic antidepressants carry a higher anticholinergic burden than SSRIs and may therefore confer greater risk, although direct comparative data remain limited [28].

#### 3.2.3. Triptans

Triptans are thought to act as 5-HT_1B/1D_ agonists and produce cerebral and peripheral vasoconstriction. Their suggested association with AACG is supported mainly by case reports and small case series; robust population-level data comparable to those available for antidepressants are lacking [6,7,8,9]. Proposed mechanisms include serotonergic mydriatic and vasogenic effects triggering pupillary block in anatomically predisposed eyes, often during acute migraine treatment [30,31]. Because migraine prodromal symptoms such as headache and visual disturbance overlap with AACG features, recognition of a concurrent ocular emergency may be delayed.

#### 3.2.4. Systemic Anticholinergics

Systemic anticholinergics, including bladder antimuscarinic agents (oxybutynin, tolterodine, and solifenacin), antispasmodics, antiparkinsonian medications (trihexyphenidyl and benztropine), and hyoscine-based antiemetics, can precipitate pupillary-block AACG in anatomically predisposed eyes by potentially producing pharmacologic mydriasis [32,33]. Risk is hypothesized to be amplified by polypharmacy and cumulative anticholinergic burden [34,35]. Onset typically occurs soon after treatment initiation or dose escalation, and risk is greatest in older patients with short axial length and shallow anterior chambers. First-generation antihistamines share substantial anticholinergic activity and should also be considered during medication history-taking [6,7,8,9,34].

#### 3.2.5. Adrenergic Decongestants and Sympathomimetics

Adrenergic decongestants and sympathomimetics (e.g., pseudoephedrine and phenylephrine-containing cold remedies) stimulate the iris dilator via alpha-adrenergic receptors, producing mydriasis and pupillary-block AACG, particularly under dim illumination. Furthermore, at high doses, dextromethorphan acts as a weak serotonin reuptake inhibitor [36,37]. It has been hypothesized that a rise in extracellular serotonin may stimulate 5-HT_7_ receptors to relax the iris sphincter muscle and directly increases aqueous humor production. This proposed hypersecretion model suggests potential acceleration via the activation of 5-HT_2A_ and 5-HT_2C_ receptors in the iris-ciliary body complex, which is thought to enhance ciliary body blood flow and rapidly increases IOP; however, these molecular dynamics remain incompletely established in human clinical models [36,38,39]. Because many of these agents are available without prescription, patients may not volunteer their use unless asked directly; targeted questioning is therefore important in any patient presenting with acute angle closure [6,7,8,9].

#### 3.2.6. Alpha Blockers

Urologic prescribing deserves separate emphasis. A urology-focused review noted that AACG associated with overactive-bladder antimuscarinics appears rare and largely confined to patients with untreated narrow angles, whereas a recent nationwide cohort study reported a significant, duration-dependent association between alpha-blocker therapy for benign prostatic hyperplasia and incident AACG [40,41]. In practice, these data support documenting any already known history of narrow angles or prior AACG and, more importantly, giving patients clear instructions to seek urgent ophthalmic assessment if symptoms of AACG develop soon after treatment initiation or dose escalation.

### 3.3. Clinical Presentation and Diagnostic Approach

Medication-induced AACG usually presents with the sudden onset of blurred vision, haloes around lights, severe ocular pain, and headache, often accompanied by systemic symptoms such as nausea and vomiting [1,2,3]. Clinical examination typically reveals conjunctival injection, corneal edema, a markedly shallow anterior chamber, severely elevated IOP, and a mid-dilated or irregular pupil. In contrast, uveal effusion-related cases, classically induced by sulfonamide derivatives like topiramate, often manifest as a bilateral crisis characterized by an acute myopic shift and diffuse anterior chamber shallowing in the distinct absence of iris bombe [10,11,12,13,14,22].

The clinical relevance of medication-induced AACG is highlighted by a large national analysis of the FDA Adverse Event Reporting System by Aftab et al., 2024 [38], which evaluated 1629 reports of angle-closure glaucoma. The majority of these adverse events were linked to well-known medication classes, most commonly sulfonamides (*n* = 642), serotonergic agents (*n* = 318), antimuscarinics (*n* = 142), and sympathomimetics (*n* = 105), alongside less known medications, such as olanzapine and phentermine [38]. However, these pharmacovigilance counts indicate reporting patterns but cannot be used to estimate incidence because the number of exposed patients and the extent of under-reporting are unknown. Overall, a careful history of recent medication initiations, dose changes, and over-the-counter medication use is therefore essential for diagnosis.

### 3.4. Management Principles

In pupillary-block AACG, pilocarpine should be withheld when IOP is markedly elevated (>40–50 mmHg) because the ischemic iris sphincter may be unresponsive and paradoxical anterior lens displacement may worsen angle crowding. Pilocarpine may be introduced cautiously once IOP begins to fall. Definitive management is LPI, with evaluation and prophylactic treatment of the fellow eye when indicated by angle anatomy [1,2,3,42].

In effusion-related AACG, classically associated with topiramate, pilocarpine should be avoided; cycloplegics and topical corticosteroids are preferred, and angle reopening often follows medication withdrawal [6,7,8,9,10,11,12,13,14,21]. Although carbonic anhydrase inhibitors are standard therapy in many AACG presentations, oral acetazolamide has rarely been implicated in sulfonamide-related effusion syndromes; if such a mechanism is suspected, re-exposure should be avoided and treatment should be tailored to the presumed pathophysiology [13,14]. Long-term lens extraction or other angle procedures may be considered in eyes with persistent narrow angles or coexisting cataract [3,42,43].

### 3.5. Considerations in Practical Prescribing

**Figure 1:**
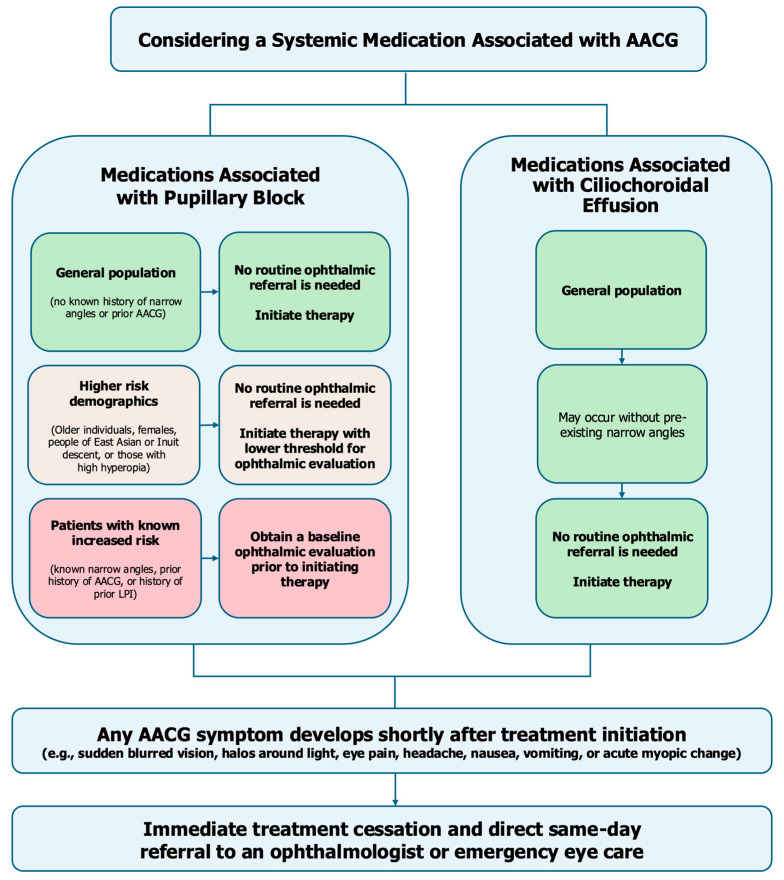
Practical pre-prescribing clinical decision-making algorithm for systemic medications implicated in acute angle-closure glaucoma (AACG). LPI, laser peripheral iridotomy.

For most prescribers, the most practical prevention strategy is not routine ophthalmic screening before treatment. These medications are not absolutely contraindicated, medication-induced AACG is rare, and a normal eye examination does not eliminate risk. Furthermore, a general history of glaucoma does not constitute a contraindication to initiating these therapies. Risk is determined primarily by anterior chamber angle anatomy rather than by the diagnosis of glaucoma itself. In particular, open-angle glaucoma, in the absence of narrow or occludable angles, is not associated with an increased risk of medication-induced AACG and should not preclude prescribing these medications. Consequently, clinical vigilance should be selectively directed toward patients with a documented history of AACG or known narrow angles, with an emphasis on providing clear counseling about warning symptoms and the need for urgent ophthalmic assessment if symptoms develop (Figure 1) [38].

A brief counseling message is usually sufficient: sudden blurred vision, halos, eye pain, headache, nausea, or vomiting soon after starting or increasing the dose of an implicated medication should prompt same-day review by an eye doctor or emergency service. Selective ophthalmology referral may be reasonable for patients with previously documented narrow angles or prior AACG, but routine gonioscopy for all patients is neither practical nor supported by current evidence. Unlike classic pupillary-block AACG in anatomically predisposed eyes, effusion-related AACG is driven by ciliochoroidal effusion and anterior displacement of the lens-iris diaphragm. It may occur in eyes without pre-existing narrow angles and is therefore not fully preventable by baseline angle screening alone. Patients with a prior LPI represent a special consideration. A patent LPI substantially reduces, but does not completely eliminate, the risk of pupillary-block AACG. Ophthalmic assessment may therefore be considered to confirm LPI patency and evaluate residual angle anatomy, particularly when the angle status is unknown. A patent LPI does not prevent ciliochoroidal effusion–related AACG.

## 4. Discussion

A critical evaluation of the medication-induced AACG literature showed varying levels of evidence quality across different medication classes. The associations documented in this review do not carry equal epidemiological weight. Sulfonamide-derived agents were supported by the relatively strongest available evidence, particularly topiramate. Similarly, the risk associated with SSRIs, SNRIs, and recent data on alpha-adrenergic antagonists is derived from large-scale, nationwide population-based cohorts and case-control studies that utilize robust exposure denominators to confirm statistical significance.

However, the evidence linking triptans and certain over-the-counter adrenergic decongestants to AACG revealed a significantly lower level of evidence, as they were largely confined to isolated clinical case reports and spontaneous adverse event entries. Because these agents are often utilized briefly or as-needed (PRN) therapies, tracking true exposure denominators in the general population is exceptionally difficult. Recognizing this hierarchy is essential for cross-specialty prescribers. While the risk associated with topiramate or chronic antidepressants warrants structured clinical awareness, the evidence for triptans remains associative and signal-based, requiring cautious clinical interpretation rather than definitive practice modifications.

The findings of this review are consistent with, and extend, earlier reviews by Wu et al., 2020 [6], Yang et al., 2019 [7], Ah-Kee et al., 2015 [8], and Lai et al., 2012 [9], which showed that a limited number of systemic medication classes account for most reported medication-induced AACG events and that these events cluster within days to weeks of treatment initiation or dose escalation [6,7,8,9]. This review expands on prior work by synthesizing recent pharmacoepidemiologic data that offer larger sample sizes and clearer exposure denominators than were available to previous authors. For instance, Na et al., 2022 [15] analyzed a nationwide Korean claims database and found that duloxetine (odds ratio [OR] = 4.04), topiramate (OR = 5.1), and sumatriptan (OR = 12.6) were each independently associated with increased odds of acute angle closure, with risk concentrated in the early exposure window [15]. By building our analysis on these modern population-level papers, this review effectively bridges literature gaps and translates risk data into a scalable counseling model tailored for cross-specialty prescribers.

Seitz et al., 2012 [17] demonstrated an increased risk (OR = 1.62) of AACG during the first 30 days after antidepressant initiation in older adults, and Chen et al., 2016 [18] reported a similar association (OR = 5.8) for SSRIs, with an increased OR reaching 8.53 for those receiving more than 20 mg daily dose in a large Taiwanese dataset [17,18]. More recently, Baek et al., 2026 [41] reported a significant, duration-dependent association between alpha-blocker use for benign prostatic hyperplasia and incident AACG, with a hazard ratio of 1.52 in a nationwide population-based cohort including 30,450 participants [41]. Case reports involving atypical antipsychotics such as olanzapine further illustrate that psychotropic-associated AACG can also occur outside antidepressant exposure and reinforce the importance of medication history and symptom counseling [29]. These estimates represent relative associations rather than absolute event probabilities. Because medication-induced AACG is rare and absolute incidence was not consistently reported across the available studies, even comparatively large odds ratios should not be interpreted as indicating a high absolute risk to an individual patient.

The mechanistic distinction between pupillary-block and uveal-effusion AACG has direct therapeutic implications yet remains under-recognized outside ophthalmology. No pretreatment strategy eliminates all risk: routine IOP measurement is insufficient because baseline pressure may be normal in anatomically vulnerable eyes, while effusion-related mechanisms such as topiramate-induced ciliochoroidal effusion are not reliably predicted by pretreatment gonioscopy. Accordingly, the most pragmatic prevention model is not universal screening but practical counseling. Patients should be informed that AACG is rare but potentially vision-threatening and that urgent ophthalmic assessment is needed if blurred vision, halos, ocular pain, headache, nausea, or vomiting develop soon after treatment initiation or dose escalation. Selective ophthalmology referral remains reasonable for patients with a known history of narrow angles, prior AACG, or a prior prophylactic iridotomy, but routine gonioscopy for all patients starting these medications is unlikely to be feasible or evidence based.

While the qualitative mechanisms of medication-induced AACG are well understood, translating this knowledge into reliable preventive frameworks requires addressing several critical evidence gaps. Incidence estimates for most implicated medications are imprecise and often unreliable, being derived from heterogeneous case series with inconsistent case definitions and poorly characterized exposure denominators [6,7,8,9,15,16,17,18,42]. Future research should be systematically directed toward the following six priorities. 

First, prospective pharmacoepidemiologic studies should be prioritized, as the current incidence data rely heavily on heterogeneous retrospective series and spontaneous case reporting. This shows underreporting and a lack of reliable exposure denominators. Therefore, large-scale, prospective multi-center cohort studies are urgently needed to establish true medication-specific incidence rates across diverse populations.

Second, standardized diagnostic criteria are needed, as the literature is currently diluted by inconsistent case definitions, where transient IOP spikes are occasionally conflated with true acute angle closure. Developing standardized consensus criteria for iatrogenic AACG will harmonize future registry data and improve reporting accuracy. Third, the clinical utility and cost-effectiveness of point-of-care, imaging-based risk stratification remain unproven. Future clinical trials should evaluate whether rapid anterior segment optical coherence tomography screening protocols in non-ophthalmic clinics can reliably identify high-risk narrow angles prior to initiating high-risk therapies.

Fourth, most of the patients exposed to these systemic medications never experience an ocular crisis, potentially pointing to an underlying genetic or biometric vulnerability. Therefore, investigating specific genetic polymorphisms associated with uveal effusion pathways or anatomical angle narrowing could yield high-value predictive biomarkers. Fifth, integrating automated electronic health record alerts that flag high-risk medications (e.g., topiramate or potent anticholinergics) when a patient has a documented diagnosis code for narrow angles or prior AACG could prevent high-risk exposures at the point of care. Finally, early symptom recognition remains the ultimate defense against permanent, medication-induced vision loss. However, validated patient-facing educational tools specifically tailored for use during fast-paced prescribing conversations have not yet been established. Developing and testing simple and clear educational materials regarding acute ocular warning signs represents a vital public health priority.

This review has limitations. Its narrative methodology, the predominance of case-based evidence, the absence of formal risk-of-bias assessment, and the inability to derive reliable medication-specific incidence estimates all limit the strength of inference. In addition, distinguishing pupillary-block from effusion-related AACG is not always straightforward in acute practice. Anterior segment optical coherence tomography and ultrasound biomicroscopy are helpful but not universally available, and treatment often begins empirically. Bilateral presentation, myopic shift, and the absence of iris bombe favor an effusion mechanism and should prompt avoidance of pilocarpine. When the mechanism remains uncertain, a cautious approach favoring cycloplegia over miotics is reasonable pending imaging confirmation.

## 5. Conclusions

Systemic medications are an uncommon but clinically important cause of AACG. The strength of evidence varies across medication classes, with the most consistent evidence supporting sulfonamide-derived agents, particularly topiramate, and population-level associations reported for several antidepressant, anticholinergic, adrenergic, and alpha-blocking agents. Because routine ophthalmic screening is neither practical nor capable of identifying all patients at risk, prevention should focus on a risk-stratified approach. Ophthalmic evaluation may be considered for patients with known narrow angles or previous AACG, while all patients prescribed implicated medications should receive brief counseling regarding warning symptoms and the need for urgent same-day ophthalmic assessment if symptoms develop. Further prospective studies are required to quantify medication-specific risks and determine whether counseling and selective referral reduce the incidence or severity of medication-induced AACG.

## Figures and Tables

**Table 1:** Summary of systemic medication classes precipitating acute angle-closure glaucoma (AACG) *.

Medication Class	Common Indications	Example Agents	Predominant Mechanism	Typical Pattern	Nature of Supporting Evidence
Sulfonamide-derived agents	Migraine; epilepsy; weight management	Topiramate; zonisamide; sulfonamide antibiotics; rarely acetazolamide and other sulfonamides	Ciliochoroidal effusion; forward lens-iris diaphragm shift; non-pupillary block	Usually bilateral; marked myopic shift; onset within days to two weeks after treatment initiation or dose increase	Systematic reviews, national adverse event database registries, and case-series data [6,7,8,9,10,11,12,13,14,15,22,25,26]
Serotonergic antidepressants (SSRIs/SNRIs) and tricyclics	Depression; anxiety; neuropathic pain	Escitalopram; sertraline; paroxetine; venlafaxine; duloxetine; amitriptyline	Mild mydriasis ± vascular effects; pupillary block in narrow angles	Often early after initiation; risk greatest in older patients with narrow angles	Large, population-based cohort and case-control studies [16,17,18,27,28,29]
Triptans	Acute migraine	Sumatriptan and other 5-HT_1B/1D_ agonists	Serotonergic vasogenic and mydriatic effects; pupillary block	Unilateral or bilateral; often within hours to days of acute migraine treatment	Case reports and small case series [6,7,8,9,30,31]
Systemic anticholinergics	Overactive bladder; motion sickness; parkinsonism; GI spasm	Oxybutynin; tolterodine; hyoscine; trihexyphenidyl; benztropine	Pharmacologic mydriasis; pupillary block	Usually early after initiation or dose increase; risk amplified by polypharmacy	Large database case-control series and medication-safety registry tracking [6,7,8,9,32,33,34,35]
Adrenergic decongestants and sympathomimetics	Nasal congestion; cold remedies	Pseudoephedrine; dextromethorphan; phenylephrine-containing cold remedies	Alpha-adrenergic stimulation of the iris dilator; pupillary block	Usually hours to days after use, especially in dim light	Adverse event databases and case reports, but lacking long-term prospective cohorts due to brief OTC use [6,7,8,9,36,37,38,39]
Alpha blockers	Benign prostatic hyperplasia; lower urinary tract symptoms	Tamsulosin; alfuzosin; doxazosin; terazosin	Chronic α1 blockade causing flaccid iris and abnormal iris/pupil physiology; pupillary block in narrow angles	Risk appears duration-dependent; consider ophthalmic assessment in anatomically predisposed patients	Large population-based cohort study data [40,41]

SSRI, selective serotonin reuptake inhibitor; SNRI, serotonin-norepinephrine reuptake inhibitor; 5-HT, 5-hydroxytryptamine; GI, gastrointestinal; OTC, over the counter. * Risk estimates were derived from heterogeneous observational designs and are not directly comparable across medication classes. They represent relative associations rather than absolute event probabilities; the absolute risk of medication-induced AACG remains low and is not reliably quantified for most agents.

## Data Availability

No new data were created or analyzed in this study. Data sharing is not applicable to this article.

## Abbreviations

The following abbreviations are used in this manuscript:

AACG	Acute Angle-Closure Glaucoma
5-HT	5-Hydroxytryptamine (Serotonin)
CNS	Central Nervous System
EAGLE	Effectiveness of Early Lens Extraction for the Treatment of Primary Angle-Closure Glaucoma (clinical trial name)
FDA	Food and Drug Administration
IOP	Intraocular Pressure
LPI	Laser Peripheral Iridotomy.
MeSH	Medical Subject Headings
OR	Odds Ratio
PACG	Primary Angle-Closure Glaucoma
SNRI	Serotonin-Norepinephrine Reuptake Inhibitor
SSRI	Selective Serotonin Reuptake Inhibitor
